# Medico-Psychology

**Published:** 1914-03-21

**Authors:** Edwin L. Ash

**Affiliations:** Director of the London Nerve Clinic (Psycho-therapeutic). 56 Seymour Street, W.


### THE EDITOR'S LETTER-BOX.

### Medico-Psychology.

*To the Editor of* The Hospital.

Sir,—Some time in the future the medical profession will realise the debt it owes to your Journal for increasing its facilities for becoming acquainted with medico-psychological problems and the work of our mental hospitals. I think I am right in saying that until you began to devote space systematically to the discussion of these questions no medical or hospital journal had ever given them the prominence they deserve. Of all questions which the medical man or hospital officer has to decide, those which he finds most difficult concern medico-psychological subjects.

It is not too much to say that there are thousands of cases dealt with in the hospitals each year in which mistakes are made not only in the receiving rooms, out-patients' departments, and wards, but in the secretary's office, owing to want of understanding of some of the elementary principles of medico-psychology. By this I by no means wish to infer that the clerks and secretaries of hospitals should undergo a course of medico-psychological training to fit them for their work. On the contrary, the onus of settling all questions of the kind must rest with the medical officers, and it is these latter who undoubtedly should be better instructed in the workings of the human mind.

At the present time it is practically impossible for the medical student to obtain adequate instruction either in the principles of normal psychology or in the application of those principles to morbid thought. There are countless instances in which eccentricities of conduct having an important bearing on questions of treatment and after-care crop up in the hospitals and utterly baffle the attempts of those who have to deal with them. Where such eccentricities pass a certain limit the unfortunate invalid becomes labelled "mental," and is either sent to the isolation wards or to the infirmary. Where his peculiarities of conduct do not appear to betoken danger to himself or others—possibly suffering mental torture beyond belief—he is either let alone, treated with some indifference, or discharged without any attempt being made to relieve his misery. There are thousands of persons suffering from obsessions, morbid fears, doubts, morbid scruples, impulses, and depressions, for example, whose mental condition closely concerns their physical health, and to whom advice as to present mode of living or future plans cannot be possibly given by doctor, nurse, or lay worker, unless the mental state is taken under careful consideration.

It is quite certain that the doctor will never be fitted to deal with his patients as fully as he should—that is to say, be able to assist them both in mind and body—until the curriculum of the medical schools include not only a course of lectures dealing with insanity, but a course specially devoted to the psychology of everyday life, the influence of suggestion, and the reality of man's inner life.

That the prominence you are giving to these subjects will hasten this much-to-be-desired innovation I have little doubt.—Believe me, yours faithfully,

Edwin L. Ash, M.D.,
Director of the London Nerve Clinic
(Psycho-therapeutic).

56 Seymour Street, W.
March 16, 1914.

